# Phosphogypsum and poultry manure enhance diversity of soil fauna, soil fertility, and barley (*Hordeum aestivum* L.) grown in calcareous soils

**DOI:** 10.1038/s41598-023-37021-3

**Published:** 2023-06-19

**Authors:** Esawy Mahmoud, Adel M. Ghoneim, Mostafa Seleem, Raghda Zuhair, Ahmed El-Refaey, Naglaa Khalafallah

**Affiliations:** 1 Department of Soil and Water Science, Faculty of Agriculture, Tanta University, Tanta, Egypt; 2 Agricultural Research Center, Field Crops Research Institute, Giza, 12112 Egypt; 3 Department of Zoology, Faculty of Science, Tanta University, Tanta, Egypt; 4 Department of Soil & Water Science, Faculty of Desert and Environmental Agriculture, Matrouh University, Mersa Matruh, Egypt

**Keywords:** Environmental chemistry, Environmental impact

## Abstract

Enrichment of calcareous soils with phosphogypsum and poultry manure amendments could increase nutrient availability, improve calcareous chemical characteristics, and enhance barley plant growth. In the current study, phosphogypsum (PG) and poultry manure (PM) were used to determine the effects of PG and PM application on soil fauna diversity, soil fertility, and barley yield. The pot experiment treatments were: C: control; PG1: 4.20 g kg^−1^ soil; PG2: 6.30 g kg^−1^ soil; PM1: 4.20 g kg^−1^ soil; PM: 6.30 g kg^−1^ soil, and their combinations. The results indicated that the application of PM alone or combined with PG had significant effects on the microbial biomass carbon (MBC), organic matter (OM), soil NPK availability, and yield of barley. Collembola and Prostigmata accounted for 50.0 and 43.3%, respectively, of the total number of soil fauna. Shannon and evenness indices increased significantly in the soil amended with PM alone or combined with PG. Amended soil with PG and/or PM significantly increased the yield and yield components of plants compared to the control. The PM1PG2 treatment increased the yield by 76.2% above the control.

## Introduction

In Egypt, the calcareous soils constitute about 27–36% of the total area. In calcareous soils where pH is high and CaCO_3_ is dominant, plants suffer from low availability of P and K; therefore, increasing the availability of these nutrients is important. Cultivation of calcareous soils presents many challenges, such as low water holding capacity, high infiltration rate, poor structure, low organic matter (OM) and clay content, low cation exchange capacity (CEC), loss of nutrients via leaching or deep percolation, surface crusting, cracking, loss of nitrogen (N) fertilizers, low availability of phosphorous (P), and micronutrients^1–4^. At present, inorganic fertilizers are being intensively used to meet the increasing demand for agricultural products in these areas, thereby threatening to contaminate groundwater as a consequence of nitrate percolation. Thus, there is a need to find an appropriate soil management strategy that could reduce inorganic fertilization application and enhance the soil fertility in calcareous soils.

Nutrient-enriched poultry manure (PM) is commonly applied as a cost-effective alternative to inorganic fertilizers. The addition of PM to calcareous soils could be a promising strategy for maintaining OM and improving soil properties^5^. However, negative environmental impacts, including greenhouse gas emissions, are generally associated with PM applications^6^. It is therefore desirable to develop strategies to reduce greenhouse gas emissions from manure-applied soils. We hypothesized that the incorporation of PM into sandy calcareous soil may be another plausible alternative to inorganic fertilizers. Phosphogypsum (PG) is a by-product resulting from the production of phosphoric acid from rock phosphate. It is the major solid waste generated from the wet process, generating approximately 5 metric tons of waste per metric ton of phosphoric acid produced^7,8^. Recycling PG is very important for producers of phosphoric acid. Based on PG chemical and physical characteristics, the potential usage is soil conditioner or landfill cover^9^. As the hazardous effects of PG waste increase in the environment owing to continued industrialization, appropriate management is required to reduce the adverse impacts on the ecosystem^10^. The major challenge in Egypt is the accumulation of PM waste, which could cause pollution issues unless environmentally and financially sustainable management strategies are used. The addition of PG to the soil is one of the most important ways to get rid of it and benefit from improving the properties of different soils and increasing crop production. It is a source of many important nutrients for plants, such as Ca, P, S, and Fe; therefore, adding PG to the soil leads to supplying plants with nutrients during the different growth stages. Phosphogypsum is also used in the reclamation of sodic soil due to its rapid solubility, the liberation of Ca in the soil matrix, and the replacement of Na, which leads to increased water infiltration in the soil^11,12^. Microbial aggregates in the soil play a role in preserving organic matter and maintaining its fertility. Microbial biomass, and enzymatic activity are important indicators of the soil for agriculture. Soil fauna is an essential component of soil health because it performs vital ecosystem functions like the decomposition of organic matter and nutrient cycling^13,14^. Soil fauna depend on organic matter and soil nutrient availability^15,16^. Soil fauna activity improves soil structure, decomposes OM, and increases soil fertility^17–19^. Orbatid mite species are utilized as bioindicators of the soil in which they dwell because they are crucial to the biogenic stage of humification of plant wastes^14^. Collembolans are regarded as environmentally friendly since they recycle and degrade agricultural waste in the soil, along with bacteria, nematodes, protozoa, and fungi^20^.

In light of these challenges and potential solutions, this study aims to evaluate the effect of PM and PG applications on some calcareous soil chemical properties and fertility. We hypothesize that adding PM and PG will improve the fertility of calcareous soils and enhance barely growth and productivity; thus, this research is significant in providing farmers with cost-effective and sustainable waste management.

The novelty of this study, PM amendment can effectively enhance soil fertility and crop production, but it cannot significantly improve the soil pH or increase soil fauna in calcareous soils. Therefore, the combination of PG and PM applications will not only lower soil pH in calcareous soils but also further improve soil fauna and increase its quality. To the best of our knowledge, there are few studies on the combination use of recycled PG with PM on improving soil quality and soil fauna in calcareous soils. Therefore, the aim of this study was to evaluate the effect of PG, PM, and their mixtures at different rates on improving soil fauna diversity, soil fertility, and the yield of barley grown in calcareous soil.

## Materials and methods

### Soil, Phosphogypsum, poultry manure characterization

The PG was obtained from a fertilizer industry factory in El-Sharkia Governorate, Egypt, while the PM was collected from a poultry farm using sawdust in the poultry litter for 40 days. A soil sample (0–20 cm) was collected from the Qetaa Maryout location (latitude 30.88°86°N, longitude 29.8°54°E), Amreya 1, Alexandria, Egypt. Organic matter (OM) in PG and PM was determined using the Walkley–Black method^21^. The pH of the soil and PG were measured in 1:2.5 (w/v ratio), and the electrical conductivity (EC) of the soil was measured in soil paste. Meanwhile, the pH and EC of PM were measured in a 1:10 (w/v) ratio using a pH meter and EC meter, respectively. Soluble calcium and magnesium in soil paste were determined using the versenate method, according to Page et al.^22^. The total N, P, and K were measured according to Sparks et al.^23^. Cation exchange capacity (CEC) was measured according to Page et al.^22^. Total calcium carbonate was determined by Collin’s Calcimeter method, Nelson^24^. Available soil P was measured using a spectrophotometer and the ascorbic acid method after the soil had been extracted with a 0.50 M NaHCO_3_ solution at a pH of 8.30^25^. Available soil N was measured using the Kjeldahel method after being extracted by a 2 M KCl solution^26^. Available soil K was determined using a flame photometer after extraction by 1.0 mol L^−1^ ammonium acetate at pH 7^27^. Microbial biomass carbon (MBC) was determined using 25 g of soil samples that were fumigated for 24 h at 250 °C with ethanol-free chloroform. After extracting the soil with K_2_SO_4_, the amount of extractable organic C was calculated using K_2_Cr_2_O_7_ and H_2_SO_4_ for 30 min at 170 °C and titrated against ferrous ammonium sulphate using ferroin as the indicator.

MBC was calculated as:

$$\mathrm{MBC}=\mathrm{ EC \,\,fumigated \,\,soil}-\mathrm{ EC\,\, unfumigated\,\, soil}/{\mathrm{K}}_{c},$$

where: EC = Extractable C; Kc = 0.38 (K_c_ is the K_2_SO_4_ extract efficiency factor^28^.

Some chemical properties of the soil, PM and PG are shown in Table 1.

**Table 1:** Selected chemical properties of the studied materials.

Properties	Units	Soil	PM	PG
pH	–	7.88 (1:2.5)	8.16 (1:10)	3.80 (1:2.5)
EC	(dS m^−1^)	11.3 ± 0.05	5.64 ± 0.07	4.05 ± 0.04
Ca^2+^	(mmol L^−1^)	57.4 ± 1.25	155 ± 1.90	29.5 ± 1.02
Mg^2+^	(mmol L^−1^)	51.4 ± 1.05	116 ± 1.63	3.00 ± 0.03
OM	(%)	0.85 ± 0.02	63.4 ± 2.05	5.40 ± 0.80
CEC	(cmolc kg^−1^)	10.6 ± 1.20	ND	58.1 ± 1.13
CaCO_3_	(g kg^−1^)	280 ± 1.25	ND	ND
Total-N	(%)	0.98 ± 0.05	4.20 ± 0.15	ND
Total-P	(%)	0.50 ± 0.01	0.68 ± 0.08	1.05 ± 0.01
Total-K	(%)	4.30 ± 0.05	1.12 ± 0.65	ND
C/N ratio	–	–	8.76	–

Each value represents a mean ± standard error (SE) of five replicates. *PM* poultry manure, *PG* phosphogypsum, *ND* not detected.

#### Plant material

Barley (*Hordeum aestivum* L.), variety 138 (origin, Egypt, 2019); *rbc*L, MW391914; *mat*K Gen Bank, MW336989; Kind, Naked; Pedigree, /5/Aths/lignee686/3/Deir Alla106//Sv.Asa/Attiki/4/Cen/Bglo.” “S”. Barley seed was brought from the barely research department, Field Crops Research Institute, Agricultural Research Center, Egypt. The collection of barley cultivar used in this experiment complies with institutional, national and international guidelines and legislation.

### Experimental set up

A greenhouse pot experiment was conducted to investigate the effects of PG and PM on some soil chemical properties and the growth of barley (*Hordeum aestivum* L.). The climatic conditions of the experimental site were: maximum and minimum temperatures of 25–30 and 9–18 °C; relative humidity of 45–60%. The treatments were: C: control, PG1: 4.20 g kg^−1^ soil; PG2: 6.30 g kg^−1^ soil; PM1: 4.20 g kg^−1^ soil; PM: 6.30 g kg^−1^ soil, and their combinations PG and PM were mixed with the soil and then packed in 10-kg plastic pots. Five seedlings of barley were planted on November 15^th^ and thinned later (after 20 days) to two plants per pot. Nitrogen fertilizer was added at a rate of 171.4 kg ha^−1^ as urea (46% N), applied in two splits after 20 and 35 days after sowing, respectively. Phosphorus and potassium fertilizers were applied at a rate of 59.5 kg ha^−1^ as super-phosphate (15%) and potassium sulphate (48% K_2_O) as basal applications before sowing. Soil moisture was maintained at field capacity (based on pot weight) by daily irrigation. Pots were arranged in a complete randomized design (CRD) with six replicates. Barley plants were harvested after 22 weeks from sowing days, and flag leaf area, yield, and its components were recorded.

### Soil fauna analysis

At the end of the experiment, soil samples were taken from each pot, and the soils were air dried before being placed in a 4 °C refrigerator. Berlese’s funnels^29^ were used to extract soil fauna and microorganisms. Separated organisms were cleaned with lactic acid and kept in 70% ethanol before being identified^27,28^. PAST, a programme for analysing paleontological data, version 4.08, was used to estimate the Shannon diversity index (H′) and Pielou’s evenness (J′). The Shannon index (H′) was derived as − Pi ln (Pi), where Pi is the ratio of the number of species i to the total numbers.

Evenness (J′) index was calculated as:

$${\mathrm{J}}^{^{\prime}}={H}^{^{\prime}}/\mathrm{lnS}.$$

According to Abdellatif et al.^30^, the soil fertility index was calculated using the following equation:

$$\mathrm{FI}={(\mathrm{FN }\times \mathrm{ FP }\times \mathrm{ FK }\times \mathrm{ FOM})}^{1/5},$$

where FI is soil fertility index; FN, FP, and FK the available soil N, P, and K and FOM for the OM (%).

### Statistical analysis

A one-way ANOVA was used to identify the differences between various treatments. Tukey post-hoc comparisons between the various groups were carried out if there were significant variations in the means**.** The obtained data were statistically analyzed using SAS 9.4 (SAS Institute Inc., Cary, NC).

## Results

### Barley yield and its components

The application of PG and PM significantly increased the spike weight, flag leaf area (FLA), 1000-grain weight, grain weight, and straw weight of the barely grown plants compared to the C treatment (Table 2). The spike weight of barley plants ranged between 14.01 and 18.38 g per pot. The addition of PM, PM2, PM1PG1, PM1PG2, PM2PG1, and PM2PG2 to the soil increased the spike weight of the barley plant by 28.89, 31.1, 12.7, 23.6, 12.4, and 19.4%, respectively, compared to the C treatment. There were no statistically significant differences in spike weight between PM1 and PM2 or between PM1PG1 and PM2PG1. The FLA of barley plants significantly increased with PM alone or combined with the PG addition (Table 2). The FLA increased by about 60.3 and 76.6% in PM1 and PM2, respectively, as compared with the C treatment. The PM2PG2 treatment increased FLA by 172.6% compared with the C treatment. Among the treatments, PM1PG2, PM2PG1, and PM2PG2, there was no significant difference. The straw weight of barley is presented in (Table 2). The effect of PM and PG applications and their combinations significantly increased the straw weight of barley. The straw weight of barley plants was reduced by 8.1% in PM2 and 18.14% in PM1, respectively, as compared to the C treatment. Between the PM2PG1 and PM2PG2 treatments, there was no statistically significant difference in straw weight.

**Table 2:** Effect of PG and PM amendments on FLA, barley yield and its components.

Treatments	Spike weight (g pot^−1^)	FLA (cm^2^)	1000-grain weight (g)	Grain weight (g pot^−1^)	Straw weight (g pot^−1^)
C	14.01 ± 0.39e	9.06 ± 1.05c	35.60 ± 1.87c	5.76 ± 0.27d	89.23 ± 0.66a
PM1	18.07 ± 0.01a	14.52 ± 1.77b	42.40 ± 1.00b	7.08 ± 0.34c	73.04 ± 0.71e
PM2	18.38 ± 0.29a	16.00 ± 0.43b	48.43 ± 1.63a	9.90 ± 0.84a	82.31 ± 0.71d
PM1PG1	15.79 ± 0.46d	16.65 ± 1.02b	44.05 ± 1.25b	9.39 ± 0.52ab	73.62 ± 1.00e
PM1PG2	17.31 ± 0.21eb	24.70 ± 2.30a	46.33 ± 1.65a	10.15 ± 0.39a	75.77 ± 0.50d
PM2PG1	15.75 ± 0.10d	24.79 ± 2.31a	48.75 ± 2.65a	9.93 ± 0.82a	78.82 ± 0.73c
PM2PG2	16.73 ± 0.07c	25.02 ± 1.50a	42.13 ± 0.99b	8.65 ± 0.36b	78.86 ± 0.36c
LSD	0.47	2.59	2.92	0.96	1.22

*C* control, *PM1* poultry manure at 4.2 g kg^−1^ soil, *PM2* poultry manure at 6.30 g kg^−1^ soil, PM1PG1: poultry manure at 4.20 g kg^−1^ soil + phosphogypsum at 4.20 g kg^−1^ soil, PM1PG2: poultry manure at 4.20 g kg^−1^ soil + phosphogypsum at 6.30 g kg^−1^ soil, PM2PG1: poultry manure at 6.30 g kg^−1^ soil + phosphogypsum at 4.20 g kg^−1^ soil, PM2PG2: poultry manure at 6.30 g kg^−1^ soil + phosphogypsum at 6.30 g kg^−1^ soil. Each value represents a means ± standard error (SE) of five replicates.

The grain weight of the barley plants ranged between 5.76 and 10.15 g pot^−1^, and the highest grain weight was observed in PM1PG2 treatment, while the lowest value was in the C treatment. The grain weight of the barley plants increased by 22.9 and by 71.8% in PM1 and PM2, respectively, as compared to the C treatment (Table 2). However, the differences in grain weight between the PM2PG1, PM2PG2, and PM2 treatments were not statistically significant.

The 1000-grain weight of barley plants increased significantly with PM alone or with the PG addition (Table 2). The 1000-grain weight of barley plants increased by 19.1 and 36.1% in PM1 and PM2 treatment, respectively, compared with the C treatment. The PM2PG1 treatment recorded the highest 1000-grain weight of barley compared to other treatments. The differences in 1000-grain weight among the PM2PG1, PM2PG2, and PM2 treatments were not statistically significant.

### Soil chemical characteristics and fertility

The added PG and PM amendments to the soil had significant effects on the calcareous soil chemical properties, i.e. soil NPK availability, OM, and MBC contents. The application of PG and PM amendments increased the soil NPK availability, OM, and MBC values significantly as compared to the C treatment (Table 3). The highest values of OM were observed in PM2PG2, while the lowest values were in the C treatment. The OM content of the soil increased by about 22.5 and 41.6% over the C in the PM1 and PM2 treatments, respectively. However, the differences in OM values between PM1PG2 and PM2PG1 were not statistically significant.

**Table 3:** Effect of PG and PM amendments on OM, MBC and soil availability of N, P and K.

Treatments	Available nutrient (mg kg^−1^)			OM (%)	MBC (mg kg^−1^)
	Soil N	Soil P	Soil K		
C	26.46 ± 0.13 g	8.62 ± 0.03f.	248.19 ± 0.03f.	1.20 ± 0.001e	2.16 ± 0.10c
PM1	31.32 ± 0.06f.	11.31 ± 005e	256.34 ± 0.02d	1.47 ± 0.003d	3.55 ± 0.45b
PM2	33.47 ± 0.22e	12.56 ± 0.02d	255.66 ± 0.31e	1.70 ± 0.002c	3.69 ± 0.13b
PM1PG1	34.36 ± 0.08d	12.64 ± 0.02d	266.92 ± 0.16b	1.53 ± 0.001d	4.13 ± 0.51b
PM1PG2	34.91 ± 0.07c	14.28 ± 0.07b	257.51 ± 0.14c	1.77 ± 0.003b	5.37 ± 0.25a
PM2PG1	35.60 ± 0.09b	13.89 ± 0.04c	269.73 ± 0.15a	1.77 ± 0.001b	4.88 ± 0.75a
PM2PG2	37.42 ± 0.16a	14.64 ± 0.05a	266.97 ± 0.02b	1.98 ± 0.001a	4.00 ± 0.22b
LSD	0.22	0.33	0.25	0.53	0.72

*C* Control, *PM1* poultry manure at 4.2 g kg^−1^ soil, *PM2* poultry manure at 6.30 g kg^−1^ soil, *PM1PG1*: poultry manure at 4.20 g kg^−1^ soil + phosphogypsum at 4.20 g kg^−1^ soil, *PM1PG2*: poultry manure at 4.20 g kg^−1^ soil + phosphogypsum at 6.30 g kg^−1^ soil, *PM2PG1*: poultry manure at 6.30 g kg^−1^ soil + phosphogypsum at 4.20 g kg^−1^ soil, *PM2PG2*: poultry manure at 6.30 g kg^−1^ soil + phosphogypsum at 6.30 g kg^−1^ soil. Each value represents a means ± standard error (SE) of five replicates.

As shown in Table 3, the MBC values were significantly increased with the addition of the PM or PG amendments as compared to the C treatment. The MBC values increased from 2.16 mg kg^−1^ in the C treatment to 5.37 mg kg^−1^ in the PM1PG2. However, the differences in MBC among the PM1, PM2, PM1PG1, and PM2PG2 were not statistically significant. Compared to the C treatment, the MBC values increased with the addition of PG and PM to the soil.

In general, the available soil NPK was significantly increased by the addition of PM alone or combined with PG amendments (Table 3). The concentrations of available soil NPK in the PM, PG, and their combinations were higher than the C treatment. Overall, the PM2PG2 treatment gave the highest soil available values of N and P, while the PM2PG1 treatment gave the highest soil available K values. Available soil N and P in PM1 and PM2 treatments were increased by 18.37%, 26.49%, 31.21%, and 45.71%, respectively, as compared to the C treatment. The addition of PM with PG increased soil available K from 248.19 to 266.92 mg kg^−1^ and to 257.51 mg kg^−1^ in PM1PG1 and PM1PG2 treatments. However, the difference in available soil P between PM2 and PM1PG1 treatment was not significant.

### Soil fauna diversity

In general, the amended soil with PG and PM had significant effects on the number of different soil microorganisms (Table 4). The increase was more evident with the higher application rates for PG and PM. Overall, the PM1PG2 treatment gave the highest total numbers of soil fauna, followed by the PM2PG1 treatment, while the lowest values were found in the C treatment. The total number of soil microorganisms increased by 166.75%, 43.75%, 337.50%, and 281.25% in P1G1, P1G2, P2G1, and P2G2, respectively, compared with the C treatment.

**Table 4:** Effect of PG and PM amendments on number of different soil microorganisms.

Treatments	Prostigmata	Mesostigmata	Orbatid mites	Collembola	Others	Total
C	1.24 ± 0.96 g	0.24 ± 0.50e	0.74 ± 0.50d	1.00 ± 1.41 g	0.74 ± 0.96a	4.01 ± 0.82f.
PM1	3.25 ± 2.06e	0 ± 0.00f.	0.25 ± 0.50f.	3.75 ± 2.06c	0.25 ± 0.50d	7.50 ± 3.87d
PM2	3.75 ± 1.50d	1.25 ± 0.50b	0.50 ± 1.50e	1.50 ± 1.71f.	0.50 ± 0.50b	7.50 ± 3.86d
PM1PG1	5.33 ± 3.06c	0.67 ± 0.01d	1.00 ± 1.73c	3.33 ± 3.06d	0.33 ± 0.58c	10.67 ± 8.08c
PM1PG2	8.75 ± 6.85a	0.25 ± 2.06e	2.25 ± 2.50b	7.25 ± 7.90a	0.75 ± 0.50a	19.4 ± 9.88a
PM2PG1	5.75 ± 1.50b	1.75 ± 1.50a	3.25 ± 1.00a	6.50 ± 1.29a	0.25 ± 0.58d	17.5 ± 4.04a
PM2PG2	8.25 ± 6.75a	0.75 ± 1.50 c	0.75 ± 0.96d	5.00 ± 2.94b	0.50 ± 0.58b	15.5 ± 5.62b

*C* Control, *PM1* poultry manure at 4.2 g kg^−1^ soil, *PM2* poultry manure at 6.30 g kg^−1^ soil; *PM1PG1*: poultry manure at 4.20 g kg^−1^ soil + phosphogypsum at 4.20 g kg^−1^ soil, *PM1PG2*: poultry manure at 4.20 g kg^−1^ soil + phosphogypsum at 6.30 g kg^−1^ soil, *PM2PG1*: poultry manure at 6.30 g kg^−1^ soil + phosphogypsum at 4.20 g kg^−1^ soil, PM2PiG2: poultry manure at 6.30 g kg^−1^ soil + phosphogypsum at 6.30 g kg^−1^ soil. Each value represents a means ± standard error (SE) of five replicates.

### Biodiversity soil indices

**Figure 1:**
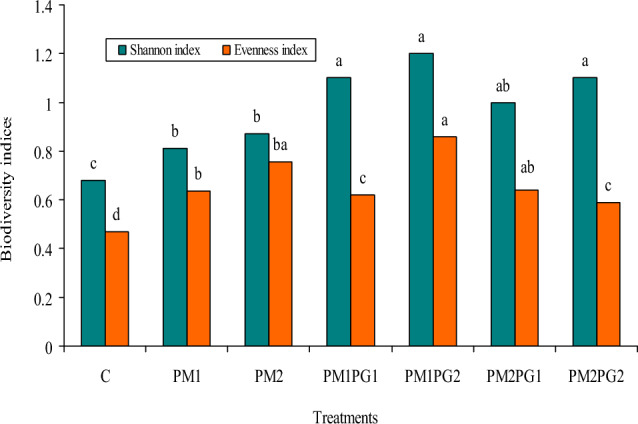
Effect of PG and PM amendments on soil diversity indices. Similar letters indicate no significant variations among treatments. *C* Control; *PM1* poultry manure at 4.2 g kg^−1^ soil; *PM2* poultry manure at 6.30 g kg^−1^ soil; *PM1PG1* poultry manure at 4.20 g kg^-1^ soil + phosphogypsum at 4.20 g kg^−1^ soil; *PM1PG2* poultry manure at 4.20 g kg^−1^ soil + phosphogypsum at 6.30 g kg^−1^ soil; *PM2PG1* poultry manure at 6.30 g kg^−1^ soil + phosphogypsum at 4.20 g kg^−1^ soil; *PM2PG2* poultry manure at 6.30 g kg^−1^ soil + phosphogypsum at 6.30 g kg^−1^ soil. Each value represents a means ± standard error (SE) of five replicates.

The PM alone or in combination with the PG treatments considerably impacted the Evenness and Shannon’s indices (Fig. 1). Overall, the PM1PG2 treatment gave the highest Evenness and Shannon's values, while the control treatment gave the lowest value. The evenness index ranged from 0.47 to 0.64 in the PM1 treatment and from 0.47 to 0.75 in the PM2 treatment. There were no statistically significant differences in the evenness index values between the PM1 and PM2PG2 or between the PM1PG2 and PM2PG2. Shannon's index values increased in the PM1 and PM2 treatments by 19.11 and 27.94%, respectively, compared to the control treatment.

## Discussion

Soil fauna contribute significantly to soil quality by improving soil structure and decomposing organic matter^31^. In this study, the total number of soil fauna, Shannon and evenness indices increased significantly when the soil was treated with PM alone or in combination with PG. Similarly, Seleem et al.^3^ found that the soil fauna populations, Shannon, and evenness indices increased significantly in calcareous soil amended by PM alone or combined with vinasse. This result may be due to the high OM content of the PM. The increasing OM content of the soil increased the diversity of the total number of Collembola and Oribatid mites^32–35^. In PM addition alone or combined with PG, there was a positive correlation between the total number of soil fauna and OM content (R^2^ = 0.82); soil fertility index (R^2^ = 0.86); MBC (R^2^ = 0.98). Islam et al.^31^ found a significant positive correlation between the soil Collembola population and organic amendments. A positive correlation between soil C and the total number of soil fauna has also been reported^31–33^. This result coincides with that obtained by Rosildaet al.^34^ who noticed that, the total numbers of Acari and Collembola species in soils increased with increasing soil OM content. Collembola represents one of the most abundant groups of soil-dwelling animals, playing a dominant role in the nutrient cycle, soil formation, and decomposition of OM^35,36^. Furthermore, the carbon content of the roots greatly affected the species formation of Oribatida mite, probably via rhizodeposition, as they feed on dead roots or root-associated fungi^37^. The increase in total number of soil fauna in soil amended by PM1PG2 treatment may be due to the higher OM and nutrient contents in the PM and PG (Table 1). Low total numbers of soil microorganisms in the C treatment may be due to the low C content^38^.

The added PG and PM amendments to the soil had significant effects on the calcareous soil chemical properties, i.e. NPK availability, OM, and MBC. The results indicated that, the type and rates of the PG and PM amendments significantly affected the availability of NPK, OM, and MBC. The PM2PG2 treatment recorded the highest OM, available N, and available P compared with other treatments. The application of the PG and PM amendments increased nutrient availability, OM, and MBC significantly as compared to the untreated soil. The increase in OM due to the application of organic amendments was confirmed in several studies^39–43^. These results may be a direct effect of PG and PM additions on increasing the nutrient contents. In addition, there is an indirect effect via improvements in soil properties that induces optimal root and shoot growth. Organic matter plays a critical role in the soil ecosystem because it provides substrates for decomposing microbes, improves soil structure, and increases water holding capacity^39,43^. Soil OM is the principal indigenous source for soil available N; beside that, it contains about 65% of the total soil P and provides significant amounts of S and other nutrients essential for plant growth^44–46^. The interaction between PM and PG led to a highly significant increase in available soil P in the soil. These findings are expected because PG contains 0.70 to 1.0% P_2_O_5_. A similar trend was obtained with the available soil N in the interaction between PG and PM treatments, as PG can reduce the loss of N as NH_3_ volatilization. Thus, more N derived from the PM was retained in the soil system, increasing the available soil N. These results may be a direct effect of PG and/or PM additions on increasing the nutrient contents. In addition, there is an indirect effect via improvements in soil properties that induces optimal root and shoot growth. The application of PM combined with PG reduces soil pH, enhances the availability of soil nutrients, and increases numbers of microorganisms. Similar results were reported by Gondek et al.^47^; Chowdhury et al.^48^ and Feng and Zhang^49^, who found that compost application increased soil OM and enhanced nutrient uptake through reduced nutrient leaching. The application of PG and PM gave the highest values of N, P, and K availability, confirming their ability to increase the efficient use of added fertilizers. However, this result may be attributed to the ability of added PG and/or PM to retain more nutrients, resulting in increased nutrient uptake. This result coincides with that obtained by Roudgarnejad et al.^50^, who postulate that the addition of different organic materials increases nutrient soil availability and uptake.

The addition of PG and/or PM significantly increased the barely yield and its components compared with the control treatment. A similar trend was reported by^51^, who reported that the application of organic amendments increased the plant height of barely planted significantly as compared to control. This may be due to the fact that the addition of PG and/or PM could increase nutrient release through direct application of amendments and/or via increasing microbial activity that decomposes OM and thus increase soil fertility^51,52^. Amended soil with PG and/or PM greatly improves plant growth by increasing nutrient availability. Similar results were reported by^53,54^, who reported that organic amendments improved the soil quality.

## Conclusions

Calcareous soils have low fertility and poor water holding capacity due to the lack of organic matter content. Phosphogypsum and poultry manure amendments significantly improved calcareous soil properties, increased the availability of NPK, microbial carbon content, and enhanced barely growth and yield. However, further field studies should be carried out to determine the proper poultry manure and phosphogypsum application rates to avoid a negative impact on the soil environment. In addition, studying the residual effect of PG and PM on yield, soil enzymes, and soil fauna diversity in long-term experiments to reduce the use of chemical fertilizers.

## Data Availability

The datasets used and analyzed during the current study are available from the corresponding author on reasonable request.
